# Th17/Treg Imbalance Reveals Local–Systemic Immune Dissociation in Nasopharyngeal Inflammation and Its Association with Endoscopic Severity

**DOI:** 10.3390/medicina62081490

**Published:** 2026-08-03

**Authors:** Manabu Mogitate

**Affiliations:** Mogitate ENT Clinic, Kawasaki 213-0011, Japan; mogitateentjp@m08.itscom.net

**Keywords:** nasopharyngeal inflammation, mucosal immunity, Th17/Treg imbalance, endoscopic severity, epipharyngeal abrasive therapy

## Abstract

*Background and Objectives:* Nasopharyngeal inflammation is difficult to assess using systemic biomarkers alone, and the diagnostic value of mucosal immune profiling remains unclear. Th17/Treg imbalance is implicated in chronic mucosal inflammation, but its local–systemic relationship in the nasopharynx has not been defined. This study aimed to characterize mucosal and peripheral immune profiles in nasopharyngeal inflammation and to evaluate their association with endoscopic severity. *Materials and Methods:* Thirty-eight patients with nasopharyngeal inflammation underwent endoscopic evaluation and immune profiling of both mucosal samples and peripheral blood. Th17 and Treg cell proportions were quantified by flow cytometry. A subset of 20 patients was reassessed after standardized epipharyngeal abrasive therapy (EAT) to evaluate systemic immune changes. *Results:* Mucosal Th17/Treg ratios were significantly higher than peripheral ratios, indicating a distinct local immune activation pattern. Mucosal Th17/Treg ratios were significantly correlated with endoscopic severity scores, whereas peripheral ratios showed no such association. Following EAT, peripheral Th17 levels decreased significantly (*p* = 0.031), whereas the Th17/Treg ratio did not change significantly. Mucosal Th17/Treg ratios also did not change significantly. *Conclusions*: Nasopharyngeal inflammation is characterized by an elevated mucosal Th17/Treg ratio that is not reflected in peripheral immune markers, suggesting compartment-specific differences between mucosal and peripheral immune responses. Mucosal Th17/Treg ratio was associated with endoscopic severity, whereas peripheral Th17 reduction was observed after EAT. Direct mucosal immune assessment may be important for accurate evaluation of local inflammatory activity and endoscopic disease severity.

## 1. Introduction

Nasopharyngeal inflammation represents a clinically underrecognized yet pathophysiologically significant condition characterized by persistent mucosal immune activation in the nasopharynx [1,2]. Although patients frequently present with nonspecific symptoms such as postnasal drip, throat discomfort, or chronic cough, these manifestations do not reliably reflect the underlying inflammatory burden. Conventional systemic biomarkers often fail to capture mucosal disease activity, highlighting a diagnostic gap in the evaluation of upper airway inflammation [3]. Recent studies have emphasized the importance of mucosal immunity in chronic inflammatory disorders, suggesting that local immune responses may diverge substantially from systemic immune profiles [4,5,6].

Among mucosal immune pathways, the balance between T helper 17 (Th17) cells and regulatory T (Treg) cells has emerged as a key determinant of chronic inflammation [7,8,9]. Th17 cells promote neutrophilic inflammation and epithelial barrier disruption, whereas Treg cells suppress excessive immune activation and maintain mucosal homeostasis. An increased Th17/Treg ratio has been implicated in various chronic inflammatory diseases [10,11]. However, despite the anatomical and immunological importance of the nasopharynx as a mucosal immune organ, the Th17/Treg balance within the nasopharyngeal microenvironment remains poorly characterized. Furthermore, the relationship between mucosal immune activation and endoscopic findings has not been systematically investigated.

Endoscopic examination of the nasopharynx provides direct visualization of mucosal inflammation, including erythema, edema, granularity, and contact bleeding. These findings are widely used in clinical practice, yet their immunological correlates are largely unknown [12,13,14]. Establishing a link between endoscopic severity and mucosal immune profiles would enhance the diagnostic value of endoscopy and provide a more objective framework for evaluating disease activity. In addition, understanding how mucosal and systemic immune responses interact—or diverge—may clarify why systemic biomarkers often fail to reflect nasopharyngeal inflammation.

Epipharyngeal abrasive therapy (EAT) is a standardized procedure that mechanically stimulates the nasopharyngeal mucosa and has been reported to modulate both local and systemic inflammation [15,16,17,18]. Recent spatial transcriptomic analysis further demonstrated that EAT can reduce residual viral RNA, suppress inflammatory signalling pathways, and restore epithelial integrity in the nasoharynx [19]. Although traditionally regarded as a therapeutic intervention, EAT also provides a unique opportunity to assess dynamic immune changes following controlled mucosal stimulation. Evaluating immune responses before and after EAT may therefore offer insight into the local–systemic immune axis and help identify biomarkers suitable for monitoring disease activity.

Therefore, the primary objective of this study was to determine whether the Th17/Treg balance differs between nasopharyngeal mucosal samples and peripheral blood in patients with chronic nasopharyngeal inflammation.

The secondary objectives were to evaluate the relationship between mucosal immune profiles and endoscopic severity and to explore longitudinal immune changes following EAT.

We hypothesized that mucosal immune alterations would be more strongly associated with local inflammatory severity than systemic immune markers. We further hypothesized that direct assessment of the nasopharyngeal mucosal immune environment may provide clinically relevant information that cannot be obtained from peripheral blood analysis alone.

By integrating endoscopic findings with paired mucosal and peripheral immune profiling, this study aimed to clarify the clinical significance of the Th17/Treg axis in chronic nasopharyngeal inflammation and to explore the relationship between local and systemic immune responses.

## 2. Materials and Methods

### 2.1. Study Design and Participants

This prospective observational study enrolled consecutive patients who visited the Mogitate ENT Clinic (Kawasaki, Japan) for the evaluation of nasopharyngeal inflammation between November 2025 and May 2026. Given the exploratory nature of this study, no formal sample size calculation was performed. Chronic nasopharyngeal inflammation was diagnosed based on characteristic endoscopic findings and the presence of contact bleeding during epipharyngeal abrasive therapy (EAT), which is the established diagnostic criterion in Japan [1,13,14].

Exclusion criteria included acute upper respiratory infection, autoimmune disease, malignancy, systemic immunosuppressive therapy, or inability to undergo endoscopic examination. Demographic data, clinical symptoms, and endoscopic findings were recorded at baseline. All participants were recruited consecutively during routine clinical practice to minimize selection bias. Baseline assessment included clinical history, symptom evaluation, endoscopic findings, and immunological profiling of both peripheral blood and nasopharyngeal mucosal samples.

#### 2.1.1. Endoscopic Evaluation

Endoscopic examination was performed using a flexible transnasal endoscope (HOYA Corporation, Tokyo, Japan; EPK-i7000 processor; VNL-1190STK scope). Under white-light imaging, mucosal redness, swelling, mucus/crust adhesion, and contact bleeding during abrasion were assessed. In addition, band-limited light (Optical Enhancement mode) was used to evaluate characteristic findings, including black spots and granularity.

Findings were scored according to the EAT Review Committee criteria (3) as follows:

0 = none,

1 = mild–moderate,

2 = severe.

The total endoscopic score was calculated as the sum of all components.

#### 2.1.2. Epipharyngeal Abrasive Therapy (EAT)

EAT was performed following standard procedures [14,15,16]. After topical anesthesia with 1% xylocaine, a cotton swab soaked in 1% zinc chloride (ZnCl_2_) was inserted transnasally to abrade the entire epipharyngeal mucosa under endoscopic visualization.

EAT was performed once weekly, and follow-up evaluations were conducted after 12 sessions. Patients who exhibited contact bleeding during abrasion were diagnosed with chronic nasopharyngeal inflammation according to the established diagnostic criteria used in Japan.

All endoscopic examinations and scoring procedures were performed by the same experienced otolaryngologist (M.M.) using predefined criteria established by the EAT Review Committee. Representative findings included mucosal redness, swelling, mucus adhesion, granularity, black spots, and contact bleeding.

### 2.2. Sample Collection

#### 2.2.1. Nasopharyngeal Abrasive Samples

Before EAT and after 12 sessions, abrasive mucosal samples were collected using a sterile cotton applicator. The cotton tip was cut and placed into a heparinized tube for flow cytometric analysis [15].

#### 2.2.2. Follow-Up Cohort

Among the 38 enrolled patients, 20 completed all 12 sessions of EAT and underwent repeat evaluation of both peripheral blood and mucosal immune parameters. Patients who did not complete follow-up were excluded from the longitudinal analysis because paired post-treatment samples were unavailable. The remaining 18 patients did not complete follow-up because of scheduling constraints, patient preference, or insufficient cellular yield in post-treatment mucosal samples.

#### 2.2.3. Peripheral Blood Samples

Peripheral blood was collected at baseline and after 12 sessions to evaluate systemic immune profiles.

### 2.3. Flow Cytometric Analysis

Two-color and multicolor flow cytometry were performed to quantify Th17 and regulatory T (Treg) cell populations in both nasopharyngeal abrasive samples and peripheral blood. Cells were stained with fluorochrome-conjugated antibodies against CD3, CD4, CD8, CD25, FOXP3, IL-17, and IFN-γ. All analyses were conducted by a single experienced operator to minimize inter-operator variability.

Definitions:

▪ Th17 cells: CD4^+^IL-17^+^IFN-γ^−^
▪ Treg cells: CD4^+^CD25^+^FOXP3^+^
▪ Th17/Treg ratio: proportion of Th17 cells ÷ proportion of Treg cells within CD4^+^ T cells

All samples were processed under standardized conditions. Compensation was performed using single-stained controls, and fluorescence minus one (FMO) controls were applied to define gating thresholds.

Lymphocytes were initially identified based on forward and side scatter properties, followed by gating of CD3^+^ T cells and subsequently CD4^+^ T cells. Th17 and Treg populations were defined within this CD4^+^ T-cell population according to the marker expression described above.

To minimize sampling variability in nasopharyngeal specimens, all mucosal samples were collected using a standardized abrasion protocol and processed immediately after collection.

Flow cytometry was performed using a BD FACSLyric system, and data were analyzed with FlowJo software. Flow-cytometric analyses were performed by SRL Inc. (Tokyo, Japan) using standardized laboratory protocols. Detailed staining conditions and instrument-specific procedures were determined according to the laboratory’s validated operating procedures.

Peripheral blood and nasopharyngeal mucosal samples were processed on the day of collection using a standardized protocol. Lymphocyte populations were identified using forward- and side-scatter characteristics, followed by sequential gating of CD3+ T cells and CD4+ T cells.

To ensure analytical consistency, all samples were analyzed using identical instrument settings throughout the study period.

### 2.4. Outcome Measures

#### 2.4.1. Primary Outcomes

▪ Th17 (%) in peripheral blood
▪ Th17/Treg ratio in nasopharyngeal abrasive cells
▪ Th17/Treg ratio in peripheral blood

#### 2.4.2. Secondary Outcomes

▪ Association between endoscopic severity score and mucosal immune parameters
▪ Changes in immune parameters after 12 sessions of EAT

### 2.5. Statistical Analysis

Continuous variables were expressed as medians with interquartile ranges (IQRs).

▪ Baseline vs. post-EAT comparisons: Wilcoxon signed-rank test
▪ Correlation analyses: Spearman’s rank correlation coefficient
▪ Between-group comparisons: Mann–Whitney U test

A *p*-value < 0.05 was considered statistically significant. Statistical analyses were performed using Python with standard scientific libraries (NumPy, SciPy, and pandas).

Additional correlation analyses were considered exploratory and were interpreted cautiously. Because of the exploratory nature of the study and the limited sample size, adjustments for multiple comparisons were not applied.

### 2.6. Ethical Approval

The study was conducted at Mogitate ENT Clinic in accordance with the Declaration of Helsinki and was approved by the Ethics Committee of Ota General Hospital (approval no. 25014). Written informed consent was obtained from all participants.

### 2.7. Patient Flow

A total of 38 patients were included in the baseline cross-sectional analysis. Among them, 20 patients completed the planned 12 sessions of EAT and underwent repeat sampling for longitudinal assessment.

## 3. Results

### 3.1. Baseline Immune Characteristics

**Figure 1:**
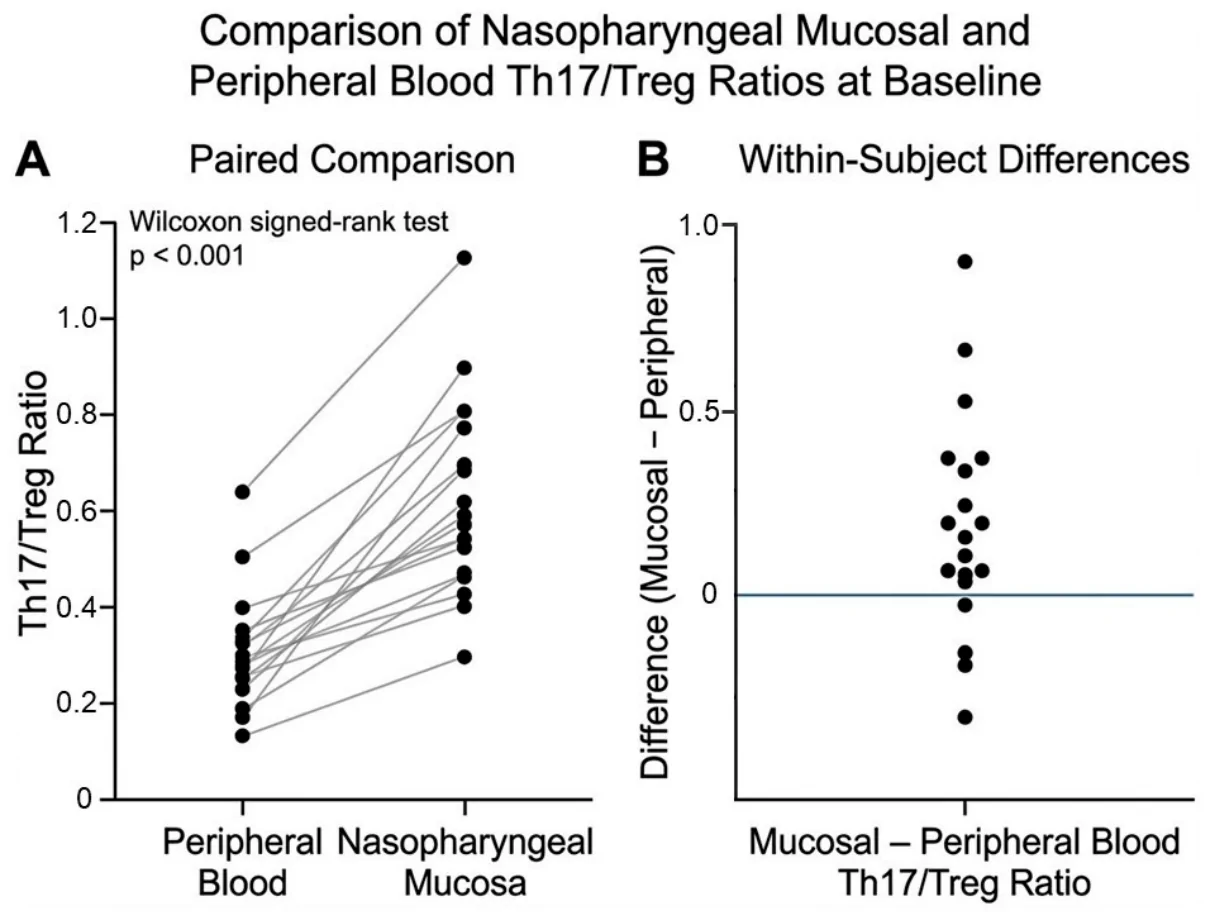
Comparison of nasopharyngeal mucosal and peripheral blood Th17/Treg ratios at baseline. (**A**) Paired comparison of Th17/Treg ratios measured in peripheral blood and nasopharyngeal mucosal samples. Each dot represents one participant, and gray lines connect paired measurements. Mucosal Th17/Treg ratios were significantly higher than peripheral blood ratios (Wilcoxon signed-rank test, *p* < 0.001). (**B**) Distribution of within-subject differences (mucosal minus peripheral blood Th17/Treg ratio). Positive values indicate higher Th17/Treg ratios in the nasopharyngeal mucosa than in peripheral blood.

At baseline, the Th17/Treg ratio in nasopharyngeal mucosal samples was significantly higher than that in peripheral blood, indicating a relatively elevated mucosal Th17/Treg ratio (Figure 1). Median Th17/Treg ratios were 0.42 (IQR: 0.28–0.61) in peripheral blood and 0.78 (IQR: 0.52–1.14) in mucosal samples. Baseline patient characteristics are summarized in Table 1.

### 3.2. Changes in Peripheral Immune Parameters After Intervention

**Figure 2:**
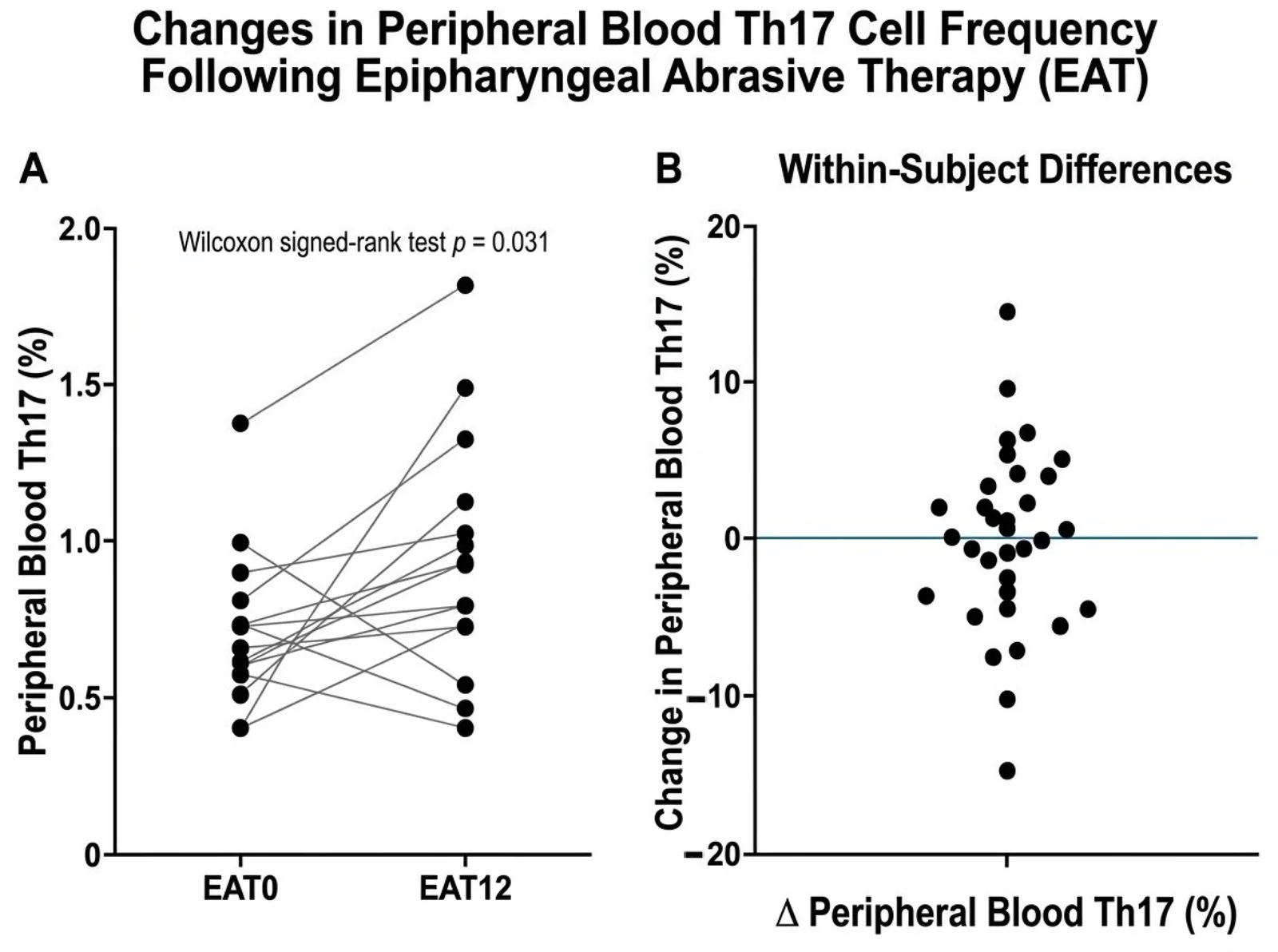
Changes in peripheral blood Th17 cell frequency following epipharyngeal abrasive therapy (EAT). (**A**) Paired peripheral blood Th17 cell frequencies before treatment (EAT0) and after 12 sessions of EAT (EAT12). Each dot represents one participant, and connecting lines indicate paired measurements. A significant reduction in peripheral blood Th17 cell frequency was observed after treatment (Wilcoxon signed-rank test, *p* = 0.031). (**B**) Distribution of within-subject changes in peripheral blood Th17 cell frequency (EAT12 − EAT0). Negative values indicate a decrease after treatment, whereas positive values indicate an increase.

**Figure 3:**
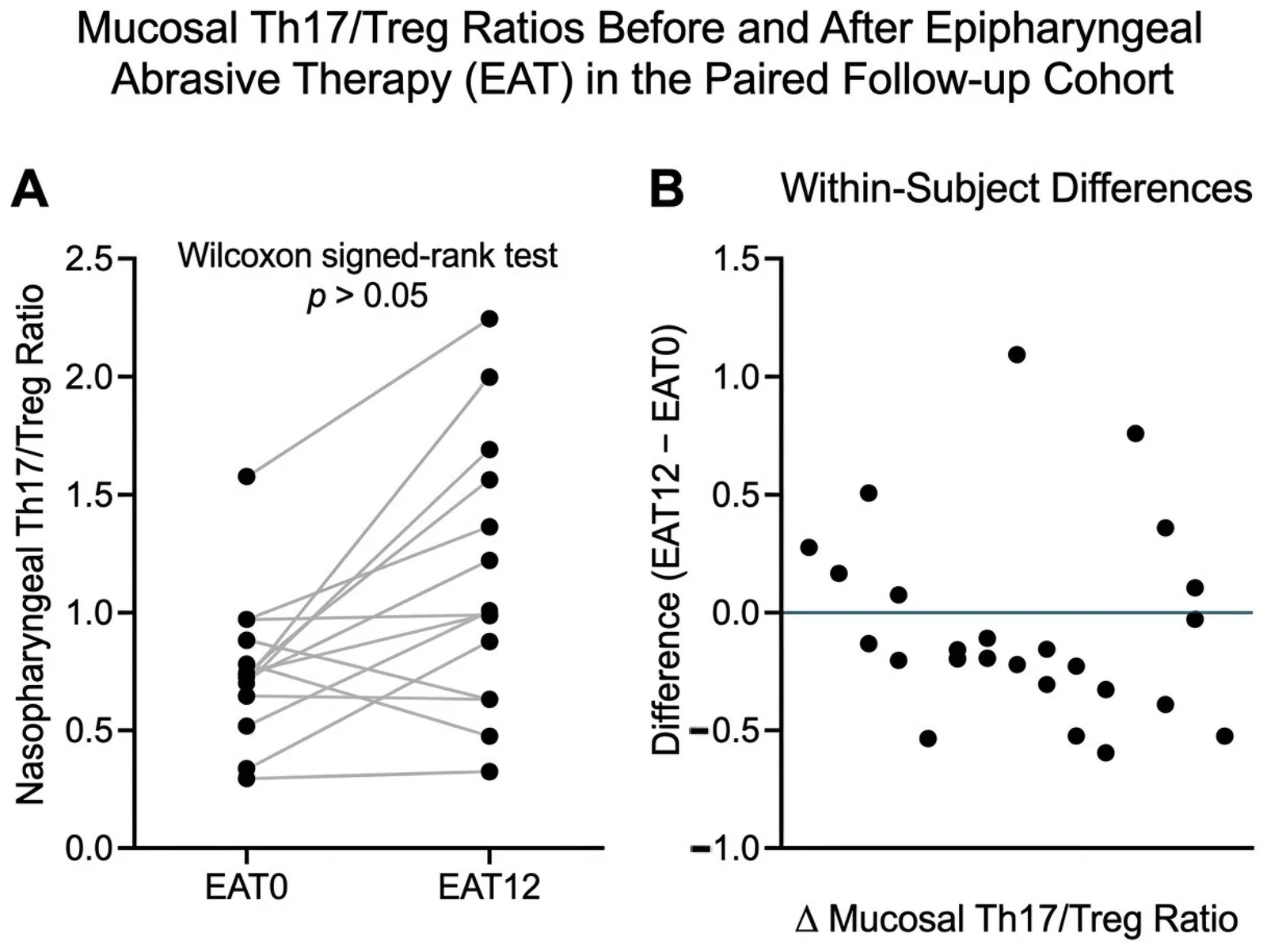
Changes in mucosal Th17/Treg ratio following epipharyngeal abrasive therapy (EAT). (**A**) Paired mucosal Th17/Treg ratios before treatment (EAT0) and after 12 sessions of EAT (EAT12). Each dot represents one participant, and connecting lines indicate paired measurements. No significant change was observed (Wilcoxon signed-rank test, *p* > 0.05). (**B**) Distribution of within-subject changes in mucosal Th17/Treg ratio (EAT12 − EAT0). Positive values indicate an increase after treatment, whereas negative values indicate a decrease.

Following the local nasopharyngeal mucosal intervention, peripheral Th17 frequencies showed a reduction (*p* = 0.031), as illustrated in Figure 2. The peripheral Th17/Treg ratio did not change significantly after treatment (Figure 3). Although most patients showed a decrease, inter-individual variability was observed. Treg frequencies exhibited a mild, non-significant increase.

### 3.3. Changes in Mucosal Immune Parameters

In contrast to peripheral blood, no significant change was observed in mucosal Th17/Treg ratios following EAT (*p* > 0.05).

### 3.4. Correlation Between Immune Changes and Clinical Findings

**Figure 4:**
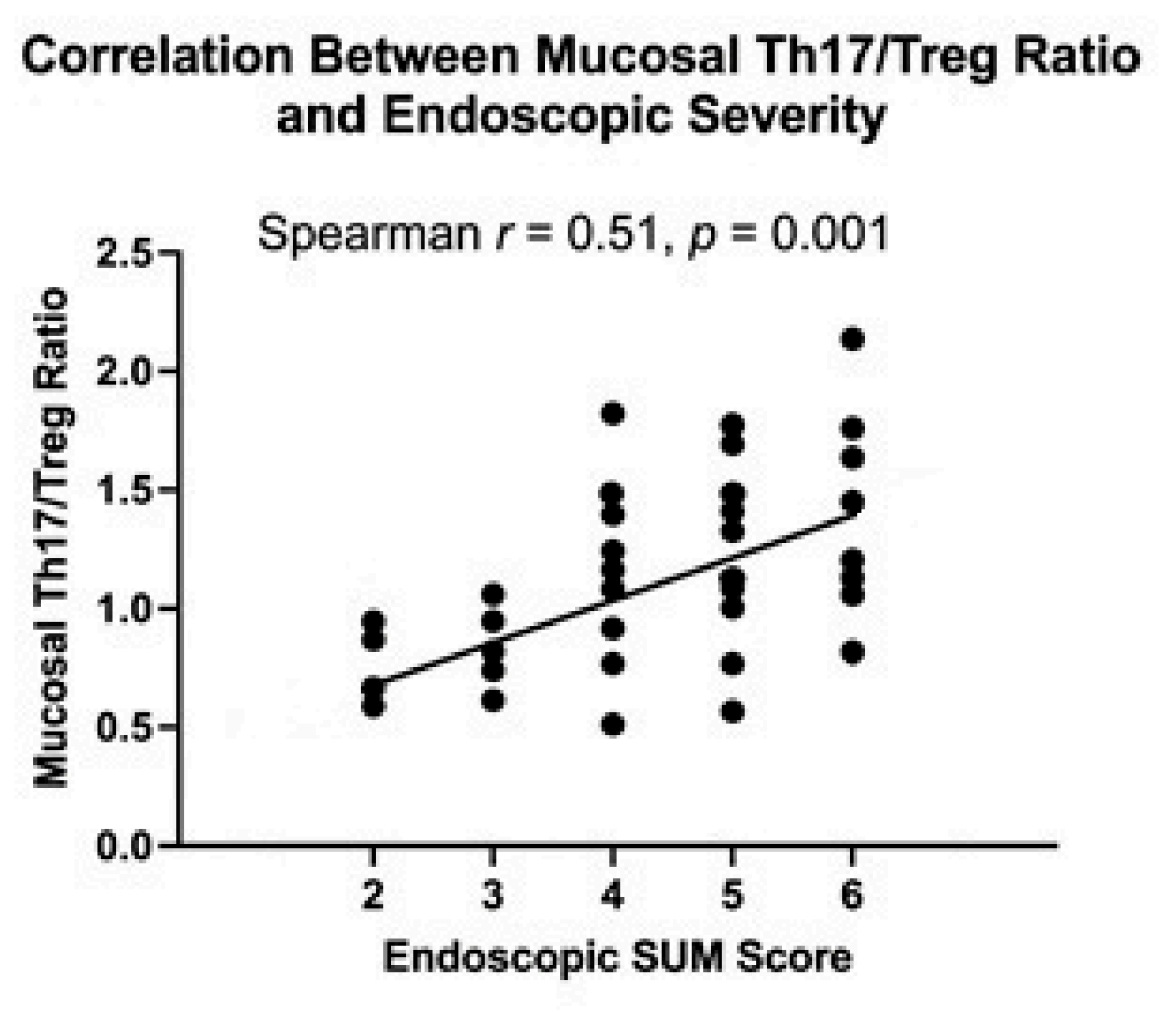
Correlation between mucosal Th17/Treg ratio and endoscopic severity score. Each dot represents one participant included in the baseline analysis. The solid line represents the fitted regression line. A significant positive correlation was observed between mucosal Th17/Treg ratio and endoscopic severity score (Spearman’s r = 0.51, *p* = 0.001).

No correlation was observed between changes in the peripheral Th17/Treg ratio and improvements in endoscopic severity scores (Spearman r = −0.07, *p* = 0.72), suggesting that peripheral immune markers may not fully reflect local mucosal inflammatory status (Figure 4). In contrast, mucosal Th17/Treg ratios were significantly correlated with endoscopic severity scores.

Additional exploratory analyses revealed further associations between systemic and mucosal immune parameters and disease features. Peripheral CD3^+^ T-cell counts showed a significant negative correlation with endoscopic severity (Spearman’s ρ = −0.42, *p* = 0.010), whereas the proportion of CD3^+^ cells among lymphocytes showed a positive correlation (ρ = 0.39, *p* = 0.017), suggesting altered systemic T-cell distribution. Blood IFNγ/IL-17-related parameters were also positively associated with endoscopic severity (*p* ≈ 0.02–0.03). In mucosal samples, IL-17-related immune profiles were significantly correlated with black spots (ρ = 0.53, *p* = 0.0048), while IFNγ-related profiles showed an inverse association (*p* = 0.011). In addition, bleeding was positively associated with mucosal lymphocyte abundance (ρ = 0.49, *p* = 0.0023) and inversely associated with regulatory T-cell levels (FOXP3^+^, ρ = −0.56, *p* = 0.0023).

## 4. Discussion

The present study shows that nasopharyngeal inflammation is characterized by an elevated local Th17/Treg ratio that is not reflected in systemic immune markers. This finding is consistent with previous observations that mucosal and peripheral immune profiles may differ across anatomical compartments, including the gut and lower airways [20,21]. A key finding of this study is the strong correlation between mucosal Th17/Treg ratios and endoscopic severity, whereas peripheral Th17/Treg ratios showed no such association. Endoscopic evaluation remains the primary practical method for assessing nasopharyngeal inflammation, and previous studies have attempted to standardize its interpretation. In particular, Ohno proposed a severity classification based on mucosal redness, swelling, postnasal drip, and crusting, demonstrating correlations between local findings and clinical symptoms [14,22,23]. However, this scoring system remains dependent on subjective visual assessment and lacks immunological validation. The present study provides biological support for endoscopic scoring by demonstrating that mucosal Th17/Treg profile correlates with disease severity, thereby linking visual findings to underlying immune mechanisms.

The Th17/Treg axis plays a central role in mucosal inflammatory regulation. Th17 cells promote neutrophilic inflammation and epithelial barrier disruption, whereas Treg cells maintain immune tolerance and suppress excessive inflammation [12,24,25,26]. An increased Th17/Treg ratio has been implicated in autoimmune and chronic inflammatory diseases, including chronic rhinosinusitis, asthma, and inflammatory bowel disease [27,28,29,30]. The plasticity and dynamic regulation of Treg cells further modulate chronic inflammatory responses [25]. The elevated mucosal Th17/Treg ratio observed in this study is consistent with these prior findings and suggests that an elevated mucosal Th17/Treg ratio is an important immunological feature of chronic nasopharyngeal inflammation.

Importantly, recent mechanistic studies support the biological relevance of epipharyngeal mucosal inflammation. Nishi et al. reported that squamous metaplasia and down-regulation of Cav1.2, ACE2, and TMPRSS2 were observed in the epipharynx after EAT [17]. In addition, reductions in residual viral RNA and inflammatory signaling pathways have been observed following EAT in spatial transcriptomic analyses [19]. These findings suggest that EAT may be associated with changes in epithelial integrity and local inflammatory pathways, although causal relationships cannot be established in observational settings.

Clinically, EAT has also been reported to improve systemic symptoms. Takezawa described a case of long-COVID-associated postural orthostatic tachycardia syndrome (POTS) that markedly improved following EAT, suggesting potential effects on autonomic function and systemic physiology [18]. Furthermore, epipharyngeal inflammation has been linked to systemic immune dysregulation through mechanisms such as the epipharynx–kidney axis and lymphoid tissue activation [8,31]. Neuro-immune modulation via vagal pathways has also been suggested as a contributor to systemic symptom improvement [28,29]. Combined with the present findings, this raises the possibility that local mucosal inflammation may be associated with systemic physiological responses through integrated immune and neural pathways. However, our results suggest that systemic immune markers may not reliably reflect local inflammatory status. Similar observations have been reported in other nasopharyngeal diseases. In nasopharyngeal carcinoma, PET-derived local metabolic parameters have been shown to predict treatment response, highlighting the clinical value of local disease assessment beyond information obtainable from systemic biomarkers alone [30].

Indeed, the present study suggests that changes in the peripheral Th17/Treg ratio did not correlate with improvements in endoscopic severity scores. This finding suggests that systemic immune responses do not necessarily reflect local mucosal recovery in the early phase after treatment. Additional exploratory analyses provide further insight into the differences between mucosal and peripheral immune responses.

Reduced peripheral CD3^+^ T-cell counts, together with an increased relative proportion of T cells, may reflect alterations in systemic T-cell distribution. Moreover, the association of IL-17-related immune profiles with black spots and the inverse association of regulatory T-cell levels with bleeding suggest a possible relationship between local immune responses and characteristic endoscopic findings.

Several factors may explain the absence of significant changes in mucosal Th17/Treg ratios after EAT. Mucosal immune environments may exhibit greater heterogeneity than peripheral blood, particularly in chronic inflammatory conditions [20,21]. In addition, abrasive sampling may capture cells from different microdomains within the nasopharynx. Furthermore, local and systemic immune responses may differ in their temporal dynamics, which could contribute to the discordance observed between mucosal and peripheral immune measurements.

This study has several limitations. First, the sample size was modest. In addition, only 20 of the 38 enrolled participants completed the follow-up assessment, and this attrition may introduce selection bias. Clinical scheduling constraints and patient preference were the primary reasons for incomplete follow-up, and therefore the longitudinal findings should be interpreted with caution. The short observation period precludes conclusions regarding long-term immune remodeling. Additionally, the study focused exclusively on the Th17/Treg axis; other immune pathways may also contribute to disease pathophysiology. In addition, contact bleeding was used both as a diagnostic finding and as a component of the endoscopic severity score, which may have introduced incorporation bias. Therefore, the observed association between mucosal immune parameters and endoscopic severity should be interpreted with caution. In addition, multiple exploratory analyses were performed without adjustment for multiple comparisons. Therefore, these findings should be considered hypothesis-generating and interpreted with caution.

Despite these limitations, the findings provide novel evidence of compartment-specific differences between mucosal and peripheral immune responses in nasopharyngeal inflammation and highlight the potential value of mucosal immune profiling. The strong association between mucosal Th17/Treg ratios and endoscopic severity underscores the importance of direct mucosal assessment.

Future studies with larger cohorts and extended follow-up are warranted to validate these findings and further clarify the mechanisms linking mucosal inflammation to systemic immune regulation. These findings may have implications for clinical decision-making, suggesting that reliance on systemic immune markers alone may lead to underestimation of disease activity.

## 5. Conclusions

This prospective observational study with paired within-subject analyses observed measurable changes in systemic immune parameters, particularly reductions in peripheral Th17 levels, after 12 sessions of EAT. These findings suggest a potential association between chronic mucosal inflammation and systemic immune responses; however, causal relationships cannot be established in this observational study.

Importantly, mucosal Th17/Treg profile was associated with endoscopic disease severity, highlighting the potential value of direct mucosal immune assessment.

Although the sample size was modest and the observation period short, the consistency of the immunological and clinical changes underscores the importance of mucosal–systemic immune interactions. Larger, controlled studies with extended follow-up are warranted to validate these findings and to further clarify the mechanisms by which local mucosal interventions influence systemic immunity. These findings suggest the potential clinical value of direct mucosal immune assessment.

## Figures and Tables

**Table 1:** Baseline characteristics of patients (*n* = 38).

Variable	Value
Age, median (IQR)	46.5 (34–57)
Sex, *n* (%)	male 9 (23.7), female 29 (76.3)
Endoscopic SUM score	5 (4–6)
Peripheral Th17/Treg ratio	0.42 (0.28–0.61)
Mucosal Th17/Treg ratio	0.78 (0.52–1.14)
Follow-up cohort (EAT12), *n* (%) 20 (52.6)	

## Data Availability

The data presented in this study are available from the corresponding author upon reasonable request.

## References

[1] Horiguchi S. (1975). The discovery of the nasopharyngitis and its influence on general diseases. Acta Otolaryngol. Suppl..

[2] Sugita R. (2010). A diagnostic method of the nasopharyngitis and a follow-up by cytology. Stomato-Pharyngol.

[3] Lundberg J.O., Weitzberg E., Nordvall S.L., Kuylenstierna R., Alving K. (1994). Primarily nasal origin of exhaled nitric oxide. Eur. Respir. J..

[4] Kuper C.F., Koornstra P.J., Hameleers D.M., Biewenga J., Spit B.J., Duijvestijn A.M., Vriesman P.J.v.B., Sminia T. (1992). The role of nasopharyngeal lymphoid tissue. Immunol. Today.

[5] Boyaka P.N., Wright P.F., Marinaro M., Kiyono H., Johnson J.E., Gonzales R.A., Ikizler M.R., Werkhaven J.A., Jackson R.J., Fujihashi K. (2000). Human nasopharyngeal-associated lymphoreticular tissues. Am. J. Pathol..

[6] Takano K., Kojima T., Go M., Murata M., Ichimiya S., Himi T., Sawada N. (2005). Dendritic cells in nasal mucosa. J. Histochem. Cytochem..

[7] Bettelli E., Carrier Y., Gao W., Korn T., Strom T.B., Oukka M., Weiner H.L., Kuchroo V.K. (2006). HLA-DR- and CD11c-positive dendritic cells penetrate beyond well-developed epithelial tight junctions in human nasal mucosa of allergic rhinitis. Nature.

[8] Noack M., Miossec P. (2014). Th17 and Treg balance in autoimmune diseases. Autoimmun. Rev..

[9] Guglani L., Khader S.A. (2010). Th17 cytokines in mucosal immunity. Curr. Opin. HIV AIDS.

[10] Miossec P., Kolls J.K. (2012). Targeting IL-17 in inflammation. Nat. Rev. Drug Discov..

[11] Kleinewietfeld M., Hafler D.A. (2013). The plasticity of human Treg and Th17 cells and its role in autoimmunity. Seminars in Immunology.

[12] Tanaka A. (2018). Band-limited light endoscopic diagnosis in chronic epipharyngitis. Stomato-Pharyngol.

[13] Mogitate M., Sasaki Y., Komiyama A. (2021). Outcome of an outpatient specialty clinic for chronic epipharyngitis. Auris Nasus Larynx.

[14] Ohno Y. (2024). Use of Nasopharyngoscopy Severity Classification of Chronic Epipharyngitis and Its Application for Evaluating the Treatment Outcomes of Epipharyngeal Abrasive Therapy. Cureus.

[15] Mogitate M. (2023). Epipharyngeal abrasive therapy downregulates CD4 cells with symptomatic recovery. Cureus.

[16] Imai K., Yamano T., Nishi S., Nishi R., Nishi T., Tanaka H., Tsunoda T., Yoshimoto S., Tanaka A., Hiromatsu K. (2022). Epipharyngeal abrasive therapy (EAT) has potential as a novel method for long COVID treatment. Viruses.

[17] Nishi K., Yoshimoto S., Nishi S., Tsunoda T., Ohno J., Yoshimura M., Hiromatsu K., Yamano T. (2022). Epipharyngeal abrasive therapy down-regulates SARS-CoV-2 entry factors ACE2 and TMPRSS2. In Vivo.

[18] Takezawa H. (2025). Successful treatment of long-COVID postural tachycardia syndrome with epipharyngeal abrasive therapy in an adolescent patient: A case report. Medicine.

[19] Nishi K., Yoshimoto S., Tanaka T., Kimura S., Tsunoda T., Watanabe A., Teranaka K., Oguma Y., Ogawa H., Kumai T. (2025). Spatial transcriptomics of the epipharynx in long COVID identifies SARS-CoV-2 signalling pathways and the therapeutic potential of epipharyngeal abrasive therapy. Sci. Rep..

[20] Kiyono H., Fukuyama S. (2004). NALT- versus Peyer’s-patch-mediated mucosal immunity. Nat. Rev. Immunol..

[21] Brandtzaeg P. (2010). Function of mucosa-associated lymphoid tissue in antibody formation. Immunol. Investig..

[22] Mogitate M., Yamaguchi Y., Fujikawa M., Fukuo A., Sasaki Y., Nishiwaki N., Ohno Y., Ito H., Watanabe Y., Wada K. (2026). Diagnostic value of black spots and granular changes in pretreatment endoscopic evaluation of chronic epipharyngitis. Auris Nasus Larynx.

[23] Mogitate M., Ito H., Ohno Y., Nishiwaki N., Yamaguchi Y., Fujikawa M., Fukuo A., Sasaki Y., Watanabe Y., Wada K. (2026). Deep Learning-Based Objective Quantification of Nasopharyngeal Endoscopic Findings for Standardized Assessment of Inflammation. Diagnostics.

[24] Korn T., Bettelli E., Oukka M., Kuchroo V.K. (2009). IL-17 and Th17 cells. Annu. Rev. Immunol..

[25] Komatsu N., Okamoto K., Sawa S., Nakashima T., Oh-hora M., Kodama T., Tanaka S., A Bluestone J., Takayanagi H. (2014). Pathogenic conversion of Foxp3^+^ T cells into TH17 cells in autoimmune arthritis. Nat. Med..

[26] Zielinski C.E., Mele F., Aschenbrenner D., Jarrossay D., Ronchi F., Gattorno M., Monticelli S., Lanzavecchia A., Sallusto F. (2012). Pathogen-induced human Th17 cells produce IFN-γ or IL-10 and are regulated by IL-1β. Nature.

[27] Hotta O., Ieiri N., Nagai M., Tanaka A., Harabuchi Y. (2022). Role of palatine tonsil and epipharyngeal lymphoid tissue in the development of glomerular active lesions in IgA nephropathy. Int. J. Mol. Sci..

[28] Ito H. (2023). The effect of epipharyngeal abrasive therapy (EAT) on the baroreceptor reflex (BR). Cureus.

[29] Ito H. (2025). Epipharyngeal abrasive therapy (EAT) field theory: A clinical framework for EAT in modulating psycho-neuro-endocrino-immune dynamics. Cureus.

[30] Quartuccio N., Sireci F., Pulizzi S., Nicolosi S., D’Oppido D., Ialuna S. (2025). Predictive Value of [18F]FDG PET/CT for Neoadjuvant Chemoradiotherapy Response in Nasopharyngeal Carcinoma. J. Clin. Med..

[31] Hotta O., Oda T. (2019). The epipharynx-kidney axis triggers glomerular vasculitis in immunoglobulin A nephropathy. Immunol. Res..

